# Commentary: Mosaic and Concerted Brain Evolution: The Contribution of Microscopic Comparative Neuroanatomy in Lower Vertebrates

**DOI:** 10.3389/fnana.2020.00006

**Published:** 2020-02-21

**Authors:** Romain Willemet

**Affiliations:** School of Life Sciences and the Environment, Royal Holloway University of London, Egham, United Kingdom

**Keywords:** adaptation, allometry, brain evolution, concerted evolution, mosaic evolution

## Introduction

In their recent Opinion article, D'Aniello et al. (2019) made the case that microscopic neuroanatomy studies in lower [*sic*] vertebrates have the potential to inform about the factors at play during brain evolution. This is a sound claim, well-illustrated by the examples chosen by the authors. However, the discussion of the existing frameworks on brain evolution is somewhat misleading, which limits the scope of this otherwise valid point.

## Context and Semantic

Finlay and Darlington (1995) found that the timing of development of brain components correlates with the relative changes in the size of brain components between species of different brain size (late equals large model). They hypothesized that this observation was indicative of a cause and effect relationship, in such a way that (i) the selection on the function(s) of one brain component would necessarily lead to whole-brain changes following these (developmentally) predetermined trajectories, and that, as such, (ii) differences in the relative size of brain components above these concerted trends between taxa and species were mostly irrelevant compared to this major developmental effect. In contrast, Barton and Harvey (2000), based on the differences in the relative size of particular brain components between taxa (i.e., grade-shifts) and the fact that functionally linked components seem to have coevolved, defended the view that mosaic changes were an important feature in brain evolution. Barton and Harvey (2000) originally did not detail how this claim could be conciliated with the concerted patterns found within taxa, but over the years the argument has been developed into the functional constraint hypothesis (Montgomery et al., 2016). This hypothesis stipulates that allometry between brain components is a consequence of selection on the function(s) of at least one component within a neural system, which leads to selection of the neural system as a whole to preserve the functional properties of this system (“more of the same” in Montgomery et al., 2016).

Thus, to call the developmental constraints model “the concerted model” is problematic because all models have to explain the concerted patterns widespread in vertebrate brain evolution. Indeed, the allometry patterns found in vertebrates are not necessarily evidence of developmental constraints. Moreover, the functional constraints model is only one variant of a model based on mosaic evolution (see below); to call it “the mosaic model,” as traditionally done, is a misleading generalization.

## Limits of the Traditional Models

Over the years, evidence has accumulated against the strong version of the developmental constraints model (see reviews by Willemet, 2013; Montgomery et al., 2016). While developmental factors are undoubtedly at play in brain evolution, individual brain components appear to have been mostly free to evolve without being systematically tied to the whole brain by way of developmental constraints.

The functional constraints hypothesis does not seem to provide a valid alternative on its own. Firstly, the assumption that allometry is mostly “more of the same” in term of function lacks theoretical and empirical support (Willemet, 2013). Moreover, the selection factors affecting the various functional systems were never detailed by the authors, making it impossible to verify that no only they correlate, but also that they select for “more of the same,” as opposed to functional changes. Finally, the hypothesis implies that brain evolution is mostly mediated by functional selection (under the constraints of energetic/physiological costs). Yet, part of the evolution of brain components may be due to selection on their structural properties (Willemet, 2013, 2019).

D'Aniello et al. seem to adhere to the traditional belief that the debate is polarized between these two hypotheses, and that, so to speak, keeping count on how many studies support one hypothesis or the other would allow to determine which constraint (functional or developmental) is more important in a given context. It is in this, the authors argue, that microscopic studies will be useful. Yet, this approach is problematic because, as seen above, neither hypothesis seems to be able to compensate for the shortcomings of the other.

## An Alternative: the Adaptationist Approach

Both the developmental and functional constraints models put, as their name implies, constraints at the basis of allometry. But what if adaptation was not just responsible for deviations from allometry, but was also the main mechanism behind allometry? In such an adaptationist approach of brain evolution, the changes in brain size and composition mainly result from the selection on the properties of individual components, rather than primarily from developmental or functional constraints. In this view, the allometries between brain components are mostly explained by the correlation between the selection pressures targeting their functional and structural properties. The developmental and functional constraints discussed above are an integral part of this framework, although their effect are limited compared to the other factors. Such an adaptationist approach gives new insights and allows to make unique predictions regarding the changes in brain size and composition between and within taxa, as well as their functional consequences (Willemet, 2019).

## Conclusion

An adaptationist framework predicts that the microanatomy studies mentioned by D'Aniello will continue to reveal cases of mosaic evolution not only in deviations from allometry and grade shifts between taxa, as predicted by the functional constraint hypothesis (Barton and Montgomery, 2018), but also in the construction of allometry itself. Whether this is the case or not, to interpret the results from such studies requires an acute examination of current frameworks on brain evolution. In this regard, an adaptationist alternative to constraint-based models seems worth considering.

## Author Contributions

The author confirms being the sole contributor of this work and has approved it for publication.

### Conflict of Interest

The author declares that the research was conducted in the absence of any commercial or financial relationships that could be construed as a potential conflict of interest.
